# Hydrothermal Synthesis of Hierarchical Ultrathin NiO Nanoflakes for High-Performance CH_4_ Sensing

**DOI:** 10.3389/fchem.2018.00194

**Published:** 2018-05-30

**Authors:** Qu Zhou, Zhaorui Lu, Zhijie Wei, Lingna Xu, Yingang Gui, Weigen Chen

**Affiliations:** ^1^College of Engineering and Technology, Southwest University, Chongqing, China; ^2^State Key Laboratory of Power Transmission Equipment & System Security and New Technology, Chongqing University, Chongqing, China

**Keywords:** hydrothermal synthesis, ultrathin NiO nanoflakes, methane, gas sensor, sensing performances

## Introduction

Methane (CH_4_), as a colorless and odorless gas, is the main component of natural gas and widely used in various industries and human daily life (Schoonbaert et al., 2015). However, the leakage of natural gas, oil and gas storage, transportation and distribution systems increase atmospheric CH_4_ concentration levels and lead to serious climate changes, which must be addressed (Zheng et al., 2017). Additionally, CH_4_ will be easy to explode in a range of concentration (5–15%). Therefore, it is necessary to develop rapid and accurate gas sensors for CH_4_ detection.

Nickel oxide NiO (Sun et al., 2014) is a significant p-type semiconductor, and has been widely used as catalyst (Yu et al., 2015), lithium-ion battery (Gu et al., 2016; Long et al., 2018), gas sensor (Wang et al., 2016; Zhou et al., 2018b), magnetic material (Cui et al., 2011), and so on. In recent years, many researchers have reported that NiO can be applied to fabricate high performance gas sensors for detecting some special gases such as hydrogen (Sta et al., 2016), NO_2_ (Hoa and El-Safty, 2011), ethanol (Miao et al., 2017), etc. Zhang et al. studied a methane gas sensor based on nickel oxide (NiO)/reduced graphene oxide (rGO) nanocomposite film, which exhibited a response of 15% toward 1000 ppm CH_4_ gas at 260°C (Zhang et al., 2016), and the sensing response of the pure NiO film sensor only was 2.5% under the same condition. Moreover, few reports about the synthesis of hierarchical NiO nanostructures and its application for CH_4_ detection was reported recently.

Thus, in this study we reported the successful synthesis of hierarchical ultrathin NiO nanoflakes and systematically researched their gas sensing properties to CH_4_. Interestingly, the proposed sensor exhibited high sensitivity, low optimal operating temperature, good linear relationship and excellent selectivity to CH_4_.

## Experimental design, materials, and methods

### Sample synthesis

All raw chemicals used for the synthesis of hierarchical ultrathin NiO nanoflakes were analytical graded and used as received without further purifications. In a typical hydrothermal procedure, 3 mmol of nickel nitrate hexahydrate Ni(NO_3_)_2_•6H_2_O and 0.200 g of polyvinylpyrrolidone (PVP) were dissolved in 100 mL DI deionized water under continuous stirring. After 15 min of rigorous stirring, a few drops of NH4OH solution were added to the resultant solution to maintain the pH = 11. The mixture was stirred vigorously for 30 min and then transferred into a Teflon-lined stainless steel autoclave, sealed and heated to 180°C for 6 h. After cooling to room temperature naturally, the product was collected by centrifugation and washed with DI water and ethanol several times, respectively, and dried at 60°C overnight. Finally, the dried power was calcined at 400°C for 2 h.

### Sample characterization

The phase structure of the prepared products were characterized by X-ray diffraction (XRD) using a Rigaku D/Max-1200X diffractometry with Cu-Kα radiation operated at 30 KV and 100 mA. The morphologies, microstructures and elemental compositions of the synthesized samples were investigated with a Nova 400 Nano field emission scanning electronic microscopy (FE-SEM), equipped with an energy dispersive X-ray spectroscopy (EDS). Gas sensors were fabricated with the side heated structure (Zhou et al., 2018c) and gas sensing properties of the obtained sensors were performed with the CGS-8 (Chemical gas sensor-8) intelligent gas sensing analysis system (Beijing Elite Tech Co., Ltd., Beijing, China).

## Results

### Materials characterization

**Figure 1:**
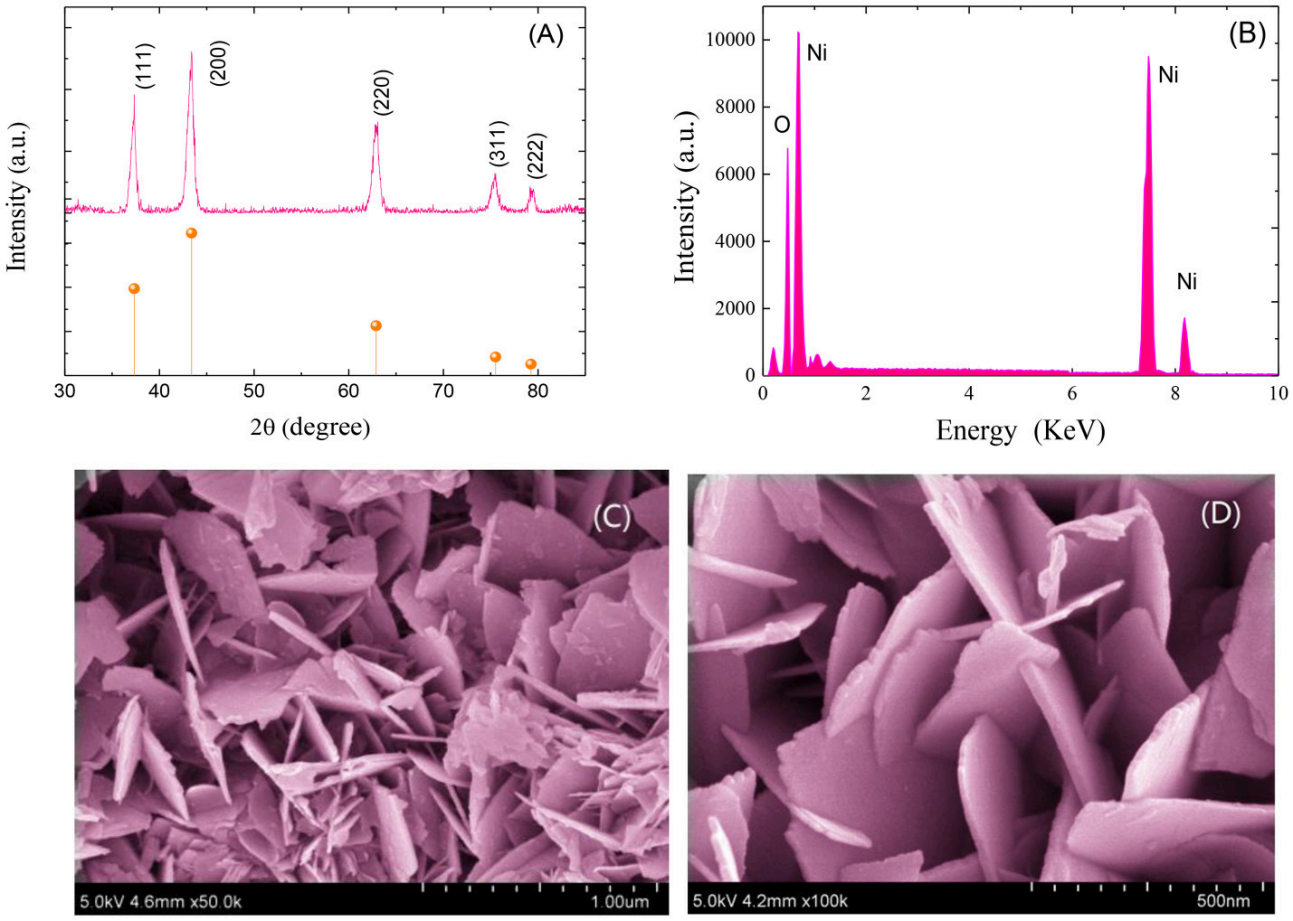
XRD patterns **(A)**, EDS patterns **(B)** and FESEM images **(C,D)** of the synthesized hierarchical ultrathin NiO nanoflakes.

Figure 1A shows the XRD pattern of the synthesized NiO sample. As shown, all the primary diffraction peaks observed at 37.30°, 43.25°, 62.85°, 75.45°, and 79.20° could be well assigned to (111), (200), (220), (311), and (222) planes of the cubic form of NiO (JCPDS Card No. 47-1049). Figure 1B depicts the EDS spectrum of the prepared NiO sample. As demonstrated, only nickel (Ni) and oxygen (O) peaks are observed with O/Ni molar ratio of nearly 1:1. No other diffraction peaks from impurities and dispersive peaks related with any element were observed, indicating a high purity of the as-prepared hierarchical ultrathin NiO nanoflakes sample.

Figures 1C,D demonstrate the FESEM image of the synthesized hierarchical NiO nanostructures, which are constructed by many ultrathin nanoflakes with smooth surface. The diameter of the NiO nanoflakes is in the scope of 300 to 400 nm with thickness ranging from 10 to 15 nm.

### Sensing performances

**Figure 2:**
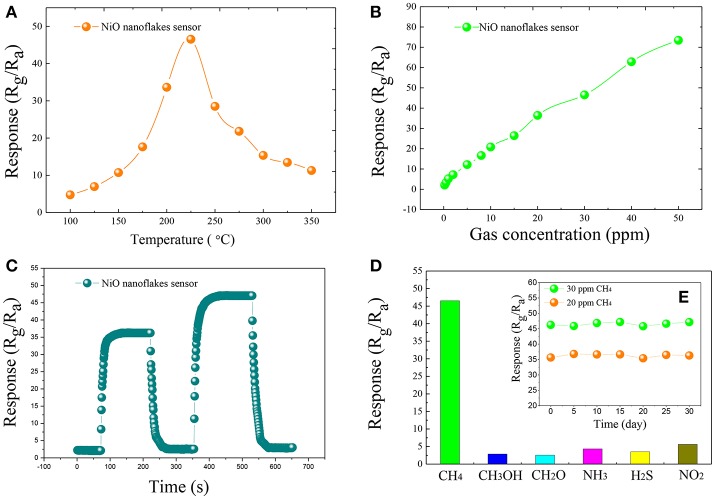
The gas response of the synthesized NiO nanoflakes sensor toward 30 ppm of CH_4_ at different working temperatures (100–350°C) **(A)**, gas response of the synthesized ultrathin NiO nanoflakes sensor to various concentration CH_4_ in the range from 0.2 to 50 ppm at 225°C **(B)**, the response and recovery curves of the synthesized ultrathin NiO nanoflakes sensor to 20 and 30 ppm CH_4_ at 225°C **(C)**, the response of NiO nanoflakes sensor toward 30 ppm of different testing gases at 225°C **(D)**, and the long-term stability of NiO nanoflakes sensor to 20 and 30 ppm CH_4_ at 225°C **(E)**.

The gas response of the fabricated side-heated sensor is defined as Rg/Ra, where Ra and Rg are the resistance values of the sensor in air and in the tested gas, respectively (Zeng et al., 2012; Zhou et al., 2018b). Figure 2A shows the relationship between the operating temperature and the gas response of the sensor to 30 ppm of CH_4_ with working temperature ranging from 100 to 350°C. As can be seen, with the increase of the temperature, the sensing response increases at first and attains its maximum value, and then decreases rapidly with further increasing temperature. The optimum operating temperature of the sensor to CH_4_ is measured to be about 225°C, lower than some already reported results (Zhang et al., 2016), and the corresponding response is 46.53.

Figure 2B illustrates the gas response of the sensor to various concentration of CH_4_ ranging from 0.2 to 50 ppm at 225°C. It is apparent that the sensing response increases rapidly with increasing gas concentration and a good linear relationship between the sensing response and gas concentration can be obtained, implying an effective candidate for low concentration CH_4_ detection.

Figure 2C demonstrates the dynamic response and recovery curve of the as-prepared sensor to 20, 30 ppm CH_4_ at 225°C. As illustrated, the sensor response increases dramatically when CH_4_ gas was injected into the test chamber, and rapidly turns back to its initial state when subjected to air for sensor recovering. The time taken by the sensor to reach 90% of the total resistance change was defined as the response (recovery) time in the case of gas adsorption (desorption). According to this definition (Li et al., 2016; Zhang et al., 2018; Zhou et al., 2018a), the response and recovery time of the NiO sensor toward 30 ppm CH4 are calculated to be about 15 s and 20 s, respectively.

Figure 2D depicts the sensing response histogram of the NiO sensor to 30 ppm of various gases, including CH_3_OH, CH_2_O, NH_3_, H_2_S, and NO_2_. It can be seen that the presented NiO sensor shows extremely high response to CH_4_ than other potential interfering gases. The long-term stability of the sensor was also measured and shown in Figure 2E, inserted in Figure 2D, where the sensor response changes slightly and keeps at a nearly constant value during the long experimental cycles, implying an excellent longtime stability and repeatability of the sensor for CH_4_ detection.

It is known to all that NiO is a typical p-type semiconducting material, and its sensing properties are predominantly controlled by the surface resistance (Wang et al., 2016). When the fabricated NiO nanoflakes sensor is exposed to air, oxygen molecules would capture free electrons to form chemisorbed oxygen in the form of ${\text{O}}_{2\text{ads}}^{-}$, ${\text{O}}_{\text{ads}}^{-}$ and ${\text{O}}_{\text{ads}}^{2-}$ absorbed on the sensor surface, increasing the number of electron holes of NiO surface and its conductivity. In CH_4_ gas ambient, oxidation-reduction reactions would take place between the pre-adsorbed oxygen ions and CH_4_ molecules, and then electrons are released back to NiO electron holes, resulting in a decreasing conductivity of the NiO nanoflakes sensor.

## Conclusions

In summary, hierarchical ultrathin NiO nanoflakes were successfully synthesized via hydrothermal process and characterized by XRD, FESEM and EDS, for the purpose of fabricating highly sensitive CH_4_ gas sensors. The diameter of the prepared NiO nanoflakes material is in the scope of 300 to 400 nm with thickness ranging from 10 to 15 nm. Side-heated gas sensor was fabricated with the synthesized ultrathin NiO nanoflakes and methane CH_4_ sensing performances were systematically evaluated. The synthesized hierarchical ultrathin NiO nanoflakes sensor exhibited high sensitivity, low optimal operating temperature, rapid response and recovery time, excellent selectivity and stability to CH_4_ gas. Moreover, good linear relationship between the sensing response and gas concentration from 0.2 to 50 ppm was also obtained. All results indicate that the synthesized hierarchical ultrathin NiO nanoflakes may be a potential sensing material for fabricating high performance gas sensor for low concentration CH_4_ detection.

## Author contributions

QZ and ZL performed the experiments and analyzed the data with the help from ZW and YG. QZ and ZL wrote the manuscript with input from all authors. All authors read and approved the manuscript.

### Conflict of interest statement

The authors declare that the research was conducted in the absence of any commercial or financial relationships that could be construed as a potential conflict of interest.
